# In vitro strategies for the enhancement of secondary metabolite production in plants: a review

**DOI:** 10.1186/s42269-022-00717-z

**Published:** 2022-02-19

**Authors:** Mohammad Afaan Fazili, Irfan Bashir, Mudasar Ahmad, Ubaid Yaqoob, Syed Naseem Geelani

**Affiliations:** 1 Plant Physiology and Biochemistry Section, Department of Botany, Aligarh Muslim University, Aligarh, UP India; 2 Plant Biotechnology and Tissue Culture Section, Department of Botany, Aligarh Muslim University, Aligarh, UP India; 3 Department of Botany, GDC Boys Pulwama, Pulwama, J&K 192301 India; 4 Department of Botany, Sri Pratap College, M. A. Road, Srinagar, J&K 190001 India; 5 Division of Social and Basic Sciences, Faculty of Forestry, SKAUST-K, Benhama, Ganderbal, J&K India

**Keywords:** In vitro, Secondary metabolites, Defense, Culture

## Abstract

**Background:**

Plants are the prime source of vital secondary metabolites (SMs) which are medicinally important for drug development, and these secondary metabolites are often used by plants in the various important tasks like defense against herbivory, interspecies defenses and against different types of stresses. For humans, these secondary metabolites are important as medicines, pigments, flavorings and drugs. Because most of the pharmaceutical industries are highly dependent on medicinal plants and their extraction, these medicinal plants are getting endangered.

**Main body:**

Plant cell culture technologies are introduced as a viable mechanism for producing and studying SMs of plants. Various types of in vitro strategies (elicitation, hairy root culture system, suspension culture system, etc.) have been considerably used for the improvement of the production of SMs of plants. For the enhancement of SM production, suspension culture and elicitation are mainly used, but hairy root culture and other organ cultures are proved to satisfy the demand of secondary metabolites. Now, it is easy to control and manipulate the pathways that produce the plant secondary metabolites.

**Conclusions:**

Techniques like plant cell, tissue and organ cultures provide a valuable method for the production of medicinally significant SMs. In recent years, most of the in vitro strategies are used due to knowledge and regulation of SM pathway in commercially valuable plants. In future, these things will provide a valuable method to sustain the feasibility of medicinal plants as the renewable sources of medicinally important compounds, and these methods will provide successful production of desired, important, valuable and also unknown compounds.

## Background

Plants are the valuable source of medicines that play a vital role in the mitigation of overall global health issues (Constabel 1990). Since from ancient times, humans’ race have relied on the traditional medicinal plants. Most of the plants synthesize large number of organic compounds which do not directly participate in the growth and development of the plant but play a significant role in plant–plant, plant–environment interaction or defensive role. These substances are called ‘secondary metabolites’ (Hussain et al. 2012). These metabolites do not participate in plant growth and development so they are produced in low quantity (Kim et al. 2002b). Secondary metabolites are used as agrochemicals, pharmaceuticals, flavor, fragrance, food additives and pesticides (Balandrin and Klocke 1988). Besides, they are performing a potent role in fighting COVID-19 (Khan et al. 2021; Hema et al. 2020). There are various roles of secondary metabolites such as protection of plants against herbivores and microbes, attracting chemicals for allelopathic agents (chemicals that influence competition among plant species), pollinators and seed-dispersing animals (Rodney et al. 2000). These are different from primary metabolites. Primary metabolites are metabolic intermediates, essential for growth and development, participated in important metabolic processes such as respiration and photosynthesis while SMs are derivatives of primary metabolites. Synthesis of primary metabolites takes place from three metabolic pathways: the Embden–Meyerhof–Parnas (EMP) pathway, hexose monophosphate (HMP) pathway and the Entner–Doudoroff pathway. SMs are synthesized either by the malonate/acetate pathway or the shikimic acid pathway (Hussain et al. 2012). The medicinal plants are getting endangered because most of the pharmaceutical industries are highly dependent on medicinal plants and their extraction. Due to their complex structure, it is difficult to synthesize these organic compounds chemically and through conventional methods. Therefore, in vitro method for production of plants SMs is the sustainable way to achieve the market demand. Besides this, plant tissue culture technique is an efficient and best alternative solution to the difficulties faced by the phytopharmaceutical industries in particular for mass propagation, germplasm conservation, study and production of biologically active compounds and for improvement of genetics. In vitro plant materials are valuable sources for the secondary metabolites production and also offer an exceptional environment for comprehensive investigation of biochemical and metabolic pathways (Mulabagal and Tsay 2004). In vitro regenerated plants provide uniform, sterile and compatible plant material. The plant materials are used in biochemical characterization and to distinguish the bioactive compounds. These compounds extracted from tissue cultures are easily purified due to uncomplicated extraction processes and absence of remarkable quantities of pigments, which probably reduce the production and processing costs. Because of these significant advances, research in this area has bloomed beyond expectations.

As plant cell, tissue and organ cultures are the sustainable ways to produce the useful secondary metabolites, but most of the experiments fail to produce the desired products. However, in many plant species, secondary metabolites production is enhanced by the callus cultures treated with elicitors, viz. salicylic acid, methyl jasmonate, chitosan and heavy metals (DiCosmo and Misawa 1985). In some plant species, secondary metabolites are also produced by hairy root culture and shooty teratoma (tumor-like) cultures. Generally, it is said that high levels of alkaloids are produced by hairy root culture (Sevon and Oksman-Caldentey 2002), whereas monoterpenes are produced by shooty teratomas (Spencer et al. 1993). However, there are a few examples of successful production of highly valuable SMs, using plant cells as factories. These include production of berberine by plant cell cultures of *Coptis japonica* and production of shikonin by cell suspension cultures of *Lithospermum erythrorhizon* (Fujita and Tabata 1986).

Field cultivation of plants to produce secondary metabolites has various difficulties (for example, low yields, instabilities in concentrations due to geographical, seasonal and environmental variations). This is why plant cell, tissue and organ cultures have been proved an appropriate alternative for the SMs production (Rao and Ravishankar 2002). Currently, different strategies have been established for synthesis of secondary compounds and the biomass accumulation such as improvement of strains, elicitation, permeabilization, optimization of media and culture environments, feeding of nutrients and precursors, biotransformation and immobilization methods. Also, in vitro studies including plant tissue culture and suspension cultures are used in different areas for the commercial production of SM (Ghorpade et al. 2011). SM production in large number has already been described in many plant cell suspension cultures (e.g., *Berberis willsoniae*, *Coleus blumei*, *Coptis japonica* and *Lithospermum erythrorhizon*), but some plant species fail to produce significant amounts of secondary metabolites in suspension culture (e.g., *Atropa belladonna*, *Duboisia leichhardtii*, *Cinchona ledgeriana*, *Digitalis lanata*) (Ellis 1988).

## Main text

### Enhancement of the secondary metabolites through plant cell culture

Plant cell and tissue cultures are another possible industrial method for the secondary metabolites production (Dicosmo and Misawa 1995). This method is self-sufficient and does not dependent on geographical or seasonal variation and accomplished by modification of different growth parameters. The idea that plant cell, tissue and organ cultures are an efficient method for the production of SMs, was introduced in late 1960. Different approaches were performed using cell culture system for the extensive production of SMs. Plant cell cultures produce SMs in different amounts and different qualities with respect to mother plants and these qualities may change with time (Tepe and Sokmen 2007). Cells are isolated from whole plant in plant cell culture, are cultured in suitable conditions and desirable product is drawn out from the cells which is cultivated. The advancements in plant tissue culture techniques provide excellent way to improve the secondary metabolites production (Chattopadhyay et al. 2002). Now, plant cell cultures play significant role in commercial production of SMs and also have advantages in cell biology, genetics and biochemistry research.

#### Suspension culture

Cell suspension culture systems are instant method for industrial application and extensive production of SMs than tissue and organ culture. This method is an ultimate and reliable source for the production of natural products (Chattopadhyay et al. 2002; Vanisree et al. 2004). It is well known that many efforts have been made for commercial production of suspension culture. In suspension cultures, required metabolites are increased, but after some time, the synthesizing capacity decreases due to insufficient nutrition or genetic dissimilarities. So, selection and preservation of high yielding cell line is very important for suspension culture (Chattopadhyay et al. 2002).

Initially, calli are generated from selected mother plants in appropriate medium which is best suited for cultivation. This appropriate medium is helpful for dedifferentiation and differentiation mechanisms. However, this task is very experiential and critical to perform, but it could be done by other way like incomplete factorial experiments or surface response methods (Hamburger and Hostettmann 1991). These generated calli are subcultured either for propagation or to induced organogenesis, embryogenesis and suspension culture (Barrales et al. 2019). For the development of suspension culture, friable part of callus transferred into liquid medium which is maintained under appropriate environments of light, temperature, agitation, aeration and other physiological parameters. Different strategies are used to develop fairly homogeneous suspension culture. Various observations show that cells in suspension cultures are highly dependent on medium combinations, callus quality, genetic variation, etc. (Chattopadhyay et al. 2002). This technique has been successfully employed in several plant species (Table 1).

**Table 1:** Secondary metabolites production through suspension culture

s. no	Plant name	Product	Results	References
1	*Taxus chinensis*	Paclitaxel	Paclitaxel production was enhanced with changing temperature from 24 to 29 °C	Choi et al. (2000)
2	*Podophyllum hexandrum*	Podophyllotoxin	Supplementation of the medium with polyvinylpyrrolidone (PVP) and pectinase increases the biomass and yield production of podophyllotoxin	Chattopadhyay et al. (2001)
3	*Bacopa monnieri*	Bacoside, saponin	Cell suspension cultures of *Bacopa monnieri* (L.) Pennell, grown in modified MS medium. Selected cell lines produced the important saponin, bacoside A, up to 1 g/100 g dry wt	Rahman et al. (2002)
4	*Tinospora cordifolia*	Protoberberine	Accumulation of berberine and jatrorrhizine (protoberberine alkaloids) was observed in both callus and cell suspension cultures. The root extracts of T. cordifolia showed higher levels of jatrorrhizine compared to the levels of berberine	Chintalwar et al. (2003)
5	*Ipomoea batatas*	Caffeoylquinic acids: caffeic acid, chlorogenic acid, 3,4-dicaffeoylquinic acid, 3,5-dicaffeoylquinic acid, 4,5-dicaffeoylquinic acid, 3,4,5-tricaffeoylquinic acid	Accumulation of phenolic compounds has been monitored in a suspension culture of anthocyanin-accumulating sweet potato cell line grown under the conditions of modified Murashige and Skoog high-anthocyanin production medium (APM) over a period of 24 days	Konczak et al. (2004)
6	*Linum album*	Podophyllotoxin, 6- methoxypodophyllotoxin	The accumulation of podophyllotoxin (PTOX) and 6-methoxypodophyllotoxin (6MPTOX) was enhanced about twofold in the suspension culture of *Linum album* line 2–5 aH following the addition of methyl jasmonate (MeJas) to the cultivation medium	van Fürden et al. (2005)
7	*Taxus baccata*	Taxol	A 2-stage suspension cell culture of *Taxus baccata* L. using modified B5 medium improved cell growth as well as productivity of secondary metabolite taxol	Khosroushahi et al. (2006)
8	*Azadirachta indica*	Azadirachtin	Glucose and phosphate were identified as the major growth-limiting nutrients during the bioreactor cultivation and production of secondary metabolites	Prakash and Srivastava, (2007)
9	*Lycopersicon esculentum*	Lycopene extraction	Optimal conditions predicted by RSM were confirmed to enhance lycopene yield from standardized tomato cell cultures by more than threefold	Lu et al. (2008)
10	*Cocos nucifera*	Phenylpropanoid	Chitosan-induced elicitation responses of dark-incubated *Cocos nucifera* (coconut) endosperm cell suspension cultures led to the rapid formation of phenylpropanoid derivatives, which essentially mimics the defense-induced biochemical changes in coconut palm as observed under in vivo conditions	Chakraborty et al. (2009)
11	*Nostoc flagelliforme*	Exopolysaccharides	The growth rate of *N. flagelliforme* cells and the accumulation of exopolysaccharides (EPS) increased prominently when NaNO_3_ and KH_2_PO_4_ were added in the liquid BG-11culture medium though phosphate had little effect on EPS yield for specific mass of cells. *N. flagelliforme* cells grew well at 25 °C and neutral pH; however, a lower or higher temperature and weak alkaline can promote EPS accumulation. With the increase of the light intensity, the growth rate of *N. flagelliforme* cells and the EPS accumulation increase accordingly	Yu et al. (2010)
12	*Arnebia hispidissima*	Alkannin	Highest alkannin content was recorded in cell suspension and callus culture established on M-9 medium. Production of alkannin was influenced by the different culture medium	Shekhawat and Shekhawat (2011)
13	*Arnebia euchroma*	Isohexenylnaphthazarins: deoxyalkannin, alkannin, hydroxyisovalerylalkannin, acetylalkannin, isobutyrylalkannin, β-2''-(S)-α-methylbutyrylalkannin, propionylalkannin, teracrylalkannin, acetylshikonin	The phytochemical investigation of the *n*-hexane extract from callus and cell suspension culture of *Arnebia euchroma* (Royle) Jonst. resulted in the isolation of number of isohexenylnaphthazarins	Damianakos et al. (2012)
14	*Stevia rebaudiana*	Stevioside	The growth kinetics of the cell suspension culture has shown a maximum specific cell growth rate of 3.26 day^−1^, doubling time of 26.35 h and cell viability of 75%, respectively. Stevioside content in cell suspension was high during exponential growth phase and decreased subsequently at the stationary phase	Mathur and Shekhawat et al. (2013)
15	*Stevia rebaudiana*	Steviol glycoside	Abiotic stress induced by the salts increased the concentration of steviol glycoside (SGs) significantly. In callus, the quantity of SGs got increased from 0.27 (control) to 1.43 and 1.57% with 0.10% NaCl, and 0.025% Na_2_CO_3_, respectively. However, in case of suspension culture, the same concentrations of NaCl and Na_2_CO_3_ enhanced the SGs content from 1.36 (control) to 2.61 and 5.14%, respectively, on the 10th day	Gupta et al. (2014)
16	*Scrophularia striata*	Acteoside	The stem explant of *S. striata* was optimum for callus induction. Modified Murashige and Skoog medium supplemented with 0.5 mg/l naphthalene acetic acid + 2.0 mg/l benzyl adenine was the most favorable medium for callus formation with the highest induction rate (100%), the best callus growth and the highest acteoside content (1.6 μg/g fresh weight)	Khanpour-Ardestani et al. (2015)
17	*Satureja khuzistanica*	Rosmarinic acid	Maximum cell fresh weight (353.5 g/L), dry weight (19.7 g/L) and rosmarinic acid (RA) production (180.0 mg/g) were attained at day 21 of culture. Cell growth and RA content were affected by nitrogen deficiency. Media containing 8.3 mM of total nitrogen (¼ of B5 standard medium) led to a minimum cell fresh weight (243.0 g/L), dry weight (17.4 g/L) and RA content (38.0 mg/g) after 21 days. The established CSC provided useful material for further optimization experiments aimed at a large-scale production of RA	Sahraroo et al. (2016)
18	*Saccharum officinarum*		Somaclonal variation occurs during the process of indirect organogenesis and RAPD and ISSR marker-based molecular analysis is a suitable method for an early detection of variation in sugarcane	Thorat et al. (2017)
19	*Salvia leriifolia*	Phenolic acids: caffeic acid, salvianolic acid B, rosmarinic acid	The maximum content of caffeic acid and salvianolic acid B were observed on the 15th day of the cultivation cycle while the highest amount of rosmarinic acid was observed on the first day. Cell cultures with 40 g/L sucrose not only produced the highest dry biomass but also the highest induction of caffeic acid and salvianolic acid B	Modarres et al. (2018)

#### Elicitation

An elicitor is a substance which initiates or improves the biosynthesis of specific compound. Many traditional approaches are proved satisfactory to increase the SMs production, but elicitation is generally one of the utmost efficacious methods. Elicitors are used in very small concentrations to a living cell system which either induces or enhances the biosynthesis of SMs (Radman et al. 2003). Elicitors can be classified according to their ‘nature’ and the ‘origin’. According to their nature, elicitors are abiotic elicitors as well as biotic elicitors, while according to their origin, elicitors are exogenous elicitors as well as endogenous elicitors. Abiotic elicitors are the non-biological substances, mostly salts of inorganic compounds and physical factors such as Cu and Cd ions, Ca^2+^ and high pH conditions, whereas biotic elicitors are biological materials. Biotic elicitors include polysaccharides (pectin, cellulose, chitin, glucans), glycoproteins, G-protein and intracellular proteins. Exogenous elicitors are those compounds which are formed exterior to the cell, e.g., polysaccharides, polyamines and fatty acids, whereas ‘endogenous elicitors’ are those types of compounds which are formed inside the cell, e.g., galacturonide or hepta-β-glucosides, etc. (Veersham 2004).

In plant tissue culture, elicitation is also done in cell suspension cultures by applying chemical or physical stresses. Stresses trigger those secondary metabolites production that are normally not formed in that plants. Now, elicitation is done with biotic elicitors (chitosan, various protein extracts, sterilized mycelium of pathogenic fungi) and abiotic factors (heavy metal salts, high and low temperature, pH, UV light, etc. Many reports are available which show that elicitors increase the quantity of SMs in plant tissue culture (Tables 2, 3). Researchers, from all over the world, have applied various types of elicitors for the improvement of SMs production in in vitro system (Sudha and Ravishankar 2003; Karuppusamy 2009). Ahmad et al. 2019 used two elicitors, chitosan and yeast extract to examine the effects on 2-hydroxy-4-methoxybenzaldehyde (2H4MB), total phenolic content (TPC), total flavonoid content (TFC) and antioxidant activity in cell suspension culture of *Decalepis salicifolia.* Chitosan was found most effective to the yeast extract at 200 µM CH and 72 h of the incubation period. It increases the1.4-fold 2H4MB in relative to control, i.e., 10.8 µg/g. Maximum content of TPC and TFC was also found, i.e., 4.8 mg/g and 4.0 mg/g, respectively. Chodisetti et al. (2013) reported positive response of *Aspergillus niger*, *Saccharomyces cerevisiae*, *Agrobacterium rhizogenes*, *Bacillus subtilis* and *Escherichia coli* extracts in the gymnemic acid production, a SM obtained from *Gymnema sylvestre*. In the cultures of plant, gymnemic acid accumulation was in the order of *A. niger*, *S. cerevisiae, A. rhizogenes, B. subtilis* and *E. coli*. Putalun et al. (2010) used other elicitors in various concentrations like methyl jasmonate 50 μM, yeast extract 0.5 mg/l and chitosan 100 mg/l for stimulation of plumbagin production in *Drosera burmanii*. The result showed that yeast extract was the most effective to enhance the plumbagin production in roots that was 3.5-fold higher than control plants. At the same way, methyl jasmonate and chitosan also gave the highest concentration in shoot and root.

**Table 2:** Abiotic elicitor and secondary metabolites production

S. no	Abiotic elicitor	Plant name	Product	References
1	Na-alginate	*Glycyrrhiza echinate*	Echinatin	Ayabe et al. (1986)
2	Metal ions: Al^3+^, Cr^3+^, Co^2+^, Ni^2+^, Cu^2+^, Zn^2+^	*Datura stramonium*	Sesquiterpenoids	Threlfall and Whitehead (1988)
3	Salicylic acid, Ca^2+^	*Daucus carota*	Chitinase	Muller et al. (1994)
4	Cu ^2+^, Cd ^2+^	*Atropa belladonna*	Tropane alkaloids	Lee et al. (1998)
5	Oxidative stress, amino acid starvation	*Arabidopsis*	Camalexin	Zhao et al. (1998)
6	Copper sulfate	*Hyoscyamus albus*	Phytoalexin	Mader (1999)
7	UV stress	*Glycyrrhiza uralensis*	Glycyrrhizin	Afreen et al. (2005)
8	Diethyl amino ethyl dichloro phenyl ether	*Catharanthus roseus*	Indole alkaloids	Lee and Rogce (2006)

**Table 3:** Biotic elicitor and secondary metabolites production

S. no	Biotic elicitor	Plant name	Product	References
1	*Ascochyta rabiei*	*Cicer arientium*	Medicarpin, Maackiain	Barz et al*.* (1988)
2	Hemicellulase	*Brugmansia candida*	Hyosujamine	Sandra et al. (1988)
3	Cellulase	*Capsicum annuum*	Capsidol	Patrica et al*.* (1996)
4	*Erwinia carotovora*	Various plant cells	Enzymes and sec. metabolites	Liu et al*.* (1998)
5	Fungal elicitor	*Cupressus lusitanica*	Indole alkaloids	Zhao et al. (2001)
6	*Trichoderma viride*	*Catharanthus roseus*	Ajmalicine	Namdeo et al. (2002)
7	Yeast elicitor	*Salvia miltiorrhiza*	Diterpenoid tanshinones	Yan et al*.* (2005)
8	Chitosan, methyl jasmonate, yeast extract	*Glycirrhiza inflata*	Glycyrrhizin	Wongwicha et al. (2011)

However, elicitation is very efficient method for enhancement of secondary metabolites but it cannot be sightlessly applied at any plant to induce metabolite production. Generally, metabolites induced or triggered by elicitation entail in plant defense system (Singh 1990). However, failure in triggering the production of SMs does not mean that the culture lacks metabolites rather continuous effects and screening are to be made. Those elicitors which are not specific to a species are not used for production of secondary metabolites because they induce ineffective elicitation. It was reported that an elicitor obtained from yeast extract could not trigger the phenylpropanoid pathway in cell suspensions of *Vanilla planifolia* (Funk and Brodelius 1990). But the same elicitor triggered the production of phytoalexin in *Glycine max* culture and alkaloid in *Thalictrum rugosum* and *Eschschottzia californica*. The pathway of phenylpropanoid could also be induced by using chitosan as an elicitor. Thus, the elicitation is an effective but challenging method and needs concentrated trial and error procedures (Singh 1990).

### Enhancement of secondary metabolites through organ culture

It is clearly known that the secondary metabolites production through cell suspension culture is not always an adequate method; so, another method which is organ culture method uses as a supernumerary method for the SMs production (Giri and Narasu 2000; Verpoorte et al. 2002; Murthy et al. 2008; Baque et al. 2012).

#### Hairy root culture

Stewart et al. (1900) firstly introduced the term ‘hairy root’ in their literature. Hairy root cultures become an advance way in the field of plant tissue and organ culture for the enhancement of SMs production. This is done by transformation of required plant species by means of *Agrobacterium rhizogenes,* natural vector system. *Agrobacterium rhizogenes* causes hairy root disease in dicotyledonous plant (Giri and Narasu 2000; Bourgaud et al. 2001). *A*. *rhizogenes,* the gram-negative soil bacterium, uses dicotyledonous plant machinery for production of its own food source (opines) for which it transformed its genes in plant’s genome. This transformation causes the development of hairy root at the infection site of host plant (Shanks and Morgan 1999). Ackermann 1977 first time used *A. rhizogenes* mediated direct transformation in higher plants. *Agrobacterium rhizogenes* and plants comprise a complex sequence for interaction which are involved in *A. tumefaciens*.

Wounded plant cells released some phenolic compounds, viz. acetosyringone and α-hydroxy acetosyringone which recognizes by *Agrobacterium* as signal molecules and attached to plants’ cell (chemotactic response). Once bacterium attached or colonized to the wounded plant cells, it leads to the insertion of T-DNA fragment of Ti-plasmid (in case of *A. tumefaciens*) or Ri-plasmid (in case of *A. rhizogenes*) to the host plant cells and integrated into the plant genome. Many vir genes located in 40-kb region of Ti-plasmid or Ri-plasmid called the virulence (vir) region play significant characters in the *Agrobacterium*-mediated transformation process. The roles of Vir have been considered in a number of outstanding review articles (Christie 1997; Gelvin 2000; Tzfira et al. 2000; Zupan et al. 2000; Tzfira and Citovsky 2000, 2002). At the site of infection, hairy root tissues or neoplastic crown gall tumor are formed by genes of T-DNA fragment. Opines synthesis facilitate the hairy root formation. Bacteria used these opines as carbon and nitrogen source to invade in the plants (Binns and Thomashow 1988). The T-DNA encoded genes are enabled to express in infected plant cells because of having eukaryotic regulatory sequences. These events of transformation are activated by vir genes, which expressed only in the presence of acetosyringone. High level of vir gene expression is induced by various sugars which act as collegial with acetosyringone. Root formation takes place at the site of infection. At the infection site, the roots development takes place as the result of T-DNA genes which coded for synthesis of auxin and other rhizogenic functions. Mostly *Agrobacterium* strains hold single type of T-DNA, but some Ri-plasmids (carrier of agropine) contain two autonomous T-DNA represented as TL-DNA (left-handed T-DNA) and TR (right-handed T-DNA). Both the TR-DNA and TL-DNA have high resemblance to the T-DNA of the Ti-plasmid of *A. tumefaciens* and Ri-plasmid of *A. rhizogenes* strains, respectively (Nilsson and Olsson 1997). The transformation and integration of TL-DNA and TR-DNA takes place separately into the genome of host plant. It was previously known that auxin synthesis encoded by the TR-DNA and TL-DNA is responsible for synthesis of a compound that persuades the infected cells to discriminate into roots under the control of endogenous auxin synthesis (Ooms et al. 1986; Shen et al. 1988). Now, it is understood that the transference of TL-DNA is important for initiation of hairy root disease while TR- DNA transfer does not incite the roots development from the transformed cultures (Nilsson and Olsson 1997; Sevon and Oksman-Caldentey 2002). The transformation ability varies related to different strains of *A. rhizogenes* (Giri et al. 1997; Kumar et al.^1991^). It is well known that hairy roots formed by various bacterial strains show various morphologies and virulence. These variations could be described by the various plasmid concealed by the strain (Nguyen et al. 1992).

*A. rhizogenes* strains were classified into two groups, according to synthesis of opine by hairy roots (Petit et al. 1983):

1. *Agropine*-type strains (e.g., A4, 15,834, HR1, LBA 9402) induce roots to produce agropine, mannopine and corresponding acids.
2. *Mannopine*-type strains (e.g., 8196, TR7, TR101) elicit roots containing only mannopine, mannopinic acid and agropinic acid.

However, Zhou et al. (1997) classified the strains of A. rhizogenes into five classes: octopine, agropine, nopaline, mannopine and cucumopine.

In plant tissue culture, hairy root culture exhibits the greatest advantage for biosynthetic capacity of SMs production related to their mother plants (Kim et al. 2002a, b; Hao et al. 2020). Hairy root cultures accomplished unlimited growth in growth hormones free culture media. It is also acknowledged that hairy root culture facilitates the production of that SM which are not present in mother plant (Veersham 2004). There are many reports which show that hairy root cultures have been established in a number of dicotyledonous and monocotyledonous for production of SMs (Mukundan et al. 1997; Doran 2002; Rudrappa et al. 2005) (Table 4). If a specific SM accumulates only in the exposed part of the plant, in that case hairy root cultures accumulate the same SM (Wallaart et al. 1999).

**Table 4:** Secondary metabolite production through hairy root cultures

S. no	Plant name	Secondary metabolite	References
1	*Brugmansia candida*	Tropane alkaloids	Spollansky et al. (2000)
2	*Glycyrrhiza glabra*	Licoagrodin	Li et al. (2000)
3	*Atropa belladonna*	Scopolamine	Bonhomme et al. (2000)
4	*Ammi majus*	Furanocoumarins (psoralen, xanthotoxine, bergapten and imperatorin)	Krolicka et al. (2001)
5	*Ophiorrhiza pumila*	Camptothecin	Sudo et al. (2002)
6	*Panax ginseng*	Ginsenosid	Palazón et al. (2003)
7	*Salvia miltiorrhiza*	Tanshinones, Tanshinone I, Tanshinone IIA, Cryptotanshinone	Zhang et al. (2004)
8	*Beta vulgaris*	Betalain	Pavlov et al. (2005)
9	*Echinacea purpurea*	Cichoric acid	Liu et al. (2006)
10	*Fagopyrum esculentum M*	Rutin	Lee et al. (2007)
11	*Silybum marianum L*	Silymarin	Rahnama et al. (2008)
12	*Rauvolfia serpentine*	Vomilenine, reserpine	Madhusudanan et al. (2008)
13	*Angelica gigas* Nakai	Pyranocoumarins	Xu et al. (2009)
14	*Arnebia hispidissima*	Shikonin	Chaudhury et al. (2010)
15	*Ophiorrhiza alata* Craib	Camptothecin	Ya-ut et al. (2011)
16	*Glycyrrhiza glabra*	Glycyrrhizin and isoliquiritigenin	Shirazi et al. (2012)
17	*Withania somnifera (L.)*	withanolide A, withanone, withaferin A	Sivanandhan et al. (2013)
18	*Tripterygium wilfordii* Hook. F	Triptolide and Wilforine	Zhu et al. (2014)
19	*Arachis hypogaea*	Resveratrol, Piceatannol, Arachidin-1, Arachidin-3	Yang et al. (2015)
20	*Linum usitatissimum*	Lignan	Gabr et al. (2016)
21	*Hyoscyamus reticulatus L*	Hyoscyamine and scopolamine	Moharrami et al. (2017)
22	*Brassica rapa* subsp. Pekinensis	Glucosinolates (GSLs)	Chung et al. (2018)
23	*Withania somnifera* L	Withaferin-A	Thilip et al. (2019)
24	*Centella asiatica*	Madecassoside, asiaticoside, madecassic acid and asiatic acid	Baek et al. (2019)

Furthermore, transformed roots have the ability to form whole viable plants and sustain their genomic stability throughout continual subculturing and plant regeneration. Additionally, a transgenic root system has incredible possibility for integrating supplementary genes along with the Ri plasmid into the host plant cells. Hairy root cultures become an appreciated means for studying the biological qualities, properties and gene expression profile of metabolic pathways. Likewise, hairy root cultures are also applied for elucidation of the intermediates and crucial enzymes participated in the biosynthesis of SM (Hu and Du 2006).

#### Shoot culture

Shoot culture is also an important method for the SMs production as hairy root cultures. It is done either to infect the aerial parts of the plants with *Agrobacterium tumefaciens* which lead to the formation of transgenic shoot (shooty teratomas) (Massot et al. 2000) or non-transgenic by the use of sufficient hormonal concentration (Saito et al. 1985). Some researchers proved that shoot cultures show nearly similar properties to hairy root cultures in production of SMs, genetic stability and relationship between growth and SMs production (Bhadra et al. 1998; Massot et al. 2000). But some differences are also existing in metabolites synthesis due to enzymes which are specially located either into roots or shoots (Subroto et al. 1996). The shooty teratomas are formed by transformation of the plant’s genetic materials with *Agrobacterium tumefaciens* nopaline or integration of *A. tumefaciens* Ti plasmid into the plant genome. Although the mechanism responsible for formation of shooty teratomas is not known, large number of plant species show the shooty teratomas formation (Hamill and Rhodes 1993). Limited number of reports are available related to growth, regeneration and application of shooty teratomas. Mainly shooty teratomas are applied in biotransformation. Saito et al. (1985) used shooty teratom as for nicotine biotransformation in *Nicotiana tabacum*. Shooty teratomas were produced in *Mentha citrate* to prove the existence of substantial amount of terpenes of mint oil (Spencer et al. 1990). In other work, Saito et al. (1991) observed that *Atropa belladonna*, *N. tabacum* and *Solarium tuberosum* synthesized tropane, nicotine and steroidal alkaloids, respectively, in shooty teratomas of. A poisonous alkaloid chemical compound, Solasodine in shooty teratomas of *Solanum eleagnifolium*, was reported by Alvarez et al. (1994). In some other plants, shooty teratomas are produced for secondary metabolites production, namely vincristine in *Catharanthus roseus* G. (Begum et al. 2009; Begum 2011), naphthoquinone in *Drosera capensis* var. *alba* (Krolicka et al. 2010).

### Enhancement of secondary metabolites through biotransformation

As it is already known that plant cell suspension cultures are valuable method for the production of important SMs (DiCosmo and Misawa 1995; Kreis and Reinhard 1989; Longo and Sanroman 2006; Smetanska 2008). But, many of the trial of plant cell suspension cultures are unable to produce the desired SMs (Kreis and Reinhard 1989; DiCosmo and Misawa 1995; Vasil et al. 1984). However, these types of cell cultures are capable to convert the exogenous compounds in a different compound with new qualities through the processes of biotransformation. Biotransformation is a process in which the main substrates are transformed in a different substrate with new characteristics through the action of suitable enzymes or microorganisms (Ye et al. 2002, 2004, 2005; Ye and Guo 2005; Zhang et al. 2007; Zhao et al. 2007). Enzymatic potential of plant cells is helpful in the process of biotransformation. These enzymes have the capacity to catalyze the various reactions like regio- and stereoselective, hydroxylation, oxido-reduction, hydrogenation, glycosylation and hydrolysis of different organic compounds as well as microorganisms (Giri et al. 2001). This is because of plant enzymes are considered as important substance for the production of specific metabolites or different compound with new qualities (Ishihara et al. 2003). Biotransformation is differing from chemical methods because there is no need for the protection of labile functional groups (Simeo and Sinisterra 2009). Biotransformation process is done in many plant species like *Eucalyptus perriniana* in which thymol, carvacrol and eugenol converted into glycosides (Shimoda et al. 2006), in tobacco hyoscyamine converted into scopolamine by the process of biotransformation (Moyano et al. 2007) and in the *Catharanthus roseus*, glycosylation biotransformation of capsaicin and 8-nordihydrocapsaicin takes place in cell cultures (Shimoda et al. 2007). Therefore, biotransformation is a technique to synthesize new active components with different qualities. Biotransformation in the cell suspension cultures of *Catharanthus roseus* and *Platycodon grandiflorum* leads to the formation of a new compound as 1b-hydroxyl desacetylcinobufagin and other unknown compound which showed cytotoxic activities against HL-60 cell lines (Ye et al. 2003).The immobilised cell techonolgy has been used in the production of secondary metabolites (Yeoman 1987). The immobilised cell techonolgy has been used in the production of secondary metabolites (Yeoman 1987).

#### Role of genetic engineering in secondary metabolite production

Genetic engineering has a prominent effect on the accumulation of secondary metabolites. Plants are capable of producing a number of chemical compounds. However, these compounds serve specific functions in the plant, and has also effects on the human body, often with positive action against diseases. Over the years, natural products from plants and their non-natural derivatives have shown to be active against different types of chronic diseases. However, isolation of such natural products can be limited due to their low bioavailability and environmental restrictions. In vitro reconstruction of plant metabolic pathways and the genetic engineering of microbes and plants have been used to generate number of secondary metabolites (Vagner and Luzia 2014). Significant advances have been made through genetic engineering of microbes and plant cells to generate a variety of compounds (e.g., isoprenoids, flavonoids or stilbenes) using a diverse array of methods to optimize these processes. These approaches have been used also to generate non-natural analogues with different bioactivities. In vitro biosynthesis allows the synthesis of intermediates as well as final products (Siebert et al. 1996, Benedito et al. 2014).

#### Micropropagation in secondary metabolites production

Plants have been used throughout the world for its medicinal powers since ancient time. The pharmacological properties of plants are based on their phytochemical components especially the secondary metabolites which are outstanding sources of value-added bioactive compounds. Secondary metabolites have complex chemical composition and are produced in response to various forms of stress to perform different physiological tasks in plants. They are used in pharmaceutical industries, cosmetics, dietary supplements, fragrances, dyes, etc. Use of these metabolites in industries has initiated a need to focus research on increasing the production by employing plant tissue culture (PTC) techniques and optimizing their large-scale production using bioreactors. PTC techniques being independent of climatic and geographical conditions will provide an incessant, sustainable, economical and viable production of secondary metabolites (Chandran et al. 2020). Different medicinal plants produce different phytoconstituents like alkaloids, flavonoids and pterocarpans. The in vitro micropropagation method can serve as a valuable method in order to produce number of secondary metabolites (Sharma et al. 2021).

#### Callus culture in secondary metabolite production

Plant growth regulators are one of the most important factors affecting cell growth, differentiation and metabolite formation. The appropriate concentration of the medium is one of the critical determinants in controlling callus growth and metabolite production. To produce secondary metabolites from medicinal plants, it is important to establish the optimal culture conditions (chemical and physical environments) for the particular plant species. Oxidative stress plays an important role in the production of secondary metabolites in plants. Phenolic compounds are considered to be secondary metabolites that are synthesized in plants through the phenylpropanoid pathway and function as a defense mechanism that reacts to various biotic and abiotic stress conditions. The exposure of plants to unfavorable conditions leads to the generation of reactive oxygen species (ROS). The well-organized way to enhance the secondary metabolite production in callus suspension cultures was deeply analyzed in *Bletilla striata* (Pan et al. 2020).

## Conclusions

Medicinal plants are important source of SMs which are directly or indirectly used by various pharmaceutical industries due to which the supply of these SMs is limited. The limited availability of biologically active, commercially valuable and medicinally important plant SMs can be overcome by using metabolic engineering and biotechnological processes. Advances in these techniques, particularly plant cell, tissue and organ cultures, provide valuable method for the production of medicinally important SMs. In cell cultures, suspension culture and elicitation are important strategy to enhance the SMs production. In various plants, these methods proved to be valuable tool for the production of SMs. Hairy root culture is an important method for enhancement of SMs production by using *Agrobacterium rhizogenes*. In some cases, hairy root cultures are considered most reliable than cell cultures for the commercial production of SMs. Shoot cultures and hairy root culture are useful because they provide a constant and reliable basis for the SMs production. The other advantage of these techniques is that they are independent of various geographical, seasonal and environmental conditions. Biotransformation helps in discovering the new compounds for pharmacological activity and for other compounds modifying their chemical structures allowing them to show pharmacological activities. In addition, biotransformation also provides synthesis of new compound in suspension culture of medicinally important plants. The use of genetic engineering, regulation of biosynthetic pathways of desired plant metabolites offers the production of commercially valuable secondary metabolites. Many molecular biology techniques, which are used in tissue cultures, induced the SM production by effecting the expression and regulation of biosynthetic pathways. In recent years, most of the in vitro strategies are used due to knowledge and regulation of SM pathway in commercially valuable plants. In future, these techniques will provide a valuable method to sustained feasibility of medicinal plant as renewable source of medicinally important compounds and these methods will provide successful production of desired, important, valuable and also unknown compounds.

## Data Availability

Not applicable.
